# AlloDerm Vs DermACELL in Breast Reconstruction: A Systematic Review and Meta-Analysis of All Head-to-Head Comparisons

**DOI:** 10.1093/asjof/ojaf084

**Published:** 2025-07-01

**Authors:** Yousef Tanas, Gioacchino De Sario Velasquez, Julie Tanas, Grace Gasper, Shadi Tanas, Keyvon Rashidi, Sarya Swed, Murad Karadsheh

## Abstract

**Background:**

AlloDerm (LifeCell, Branchburg, NJ) and DermACELL (LifeNet Health, Virginia Beach, VA) are 2 commonly used acellular dermal matrices (ADMs) in aesthetic and reconstructive breast surgery.

**Objectives:**

In this head-to-head meta-analysis, the authors aim to compare the clinical outcomes, including complication rates and BREAST-Q patient satisfaction, associated with AlloDerm and DermACELL.

**Methods:**

PubMed, Scopus, and Web of Science were searched for relevant studies in April 2024 and again in April 2025. The authors included all studies with data comparing AlloDerm and DermACELL. Statistical analyses were performed using RevMan 5.4. Heterogeneity was assessed using *I*^2^ statistics. A random-effects model was applied in case of significant heterogeneity followed by sensitivity analysis.

**Results:**

The search yielded 1006 studies, of which 14 were included in the meta-analysis. A total of 1872 patients (2940 breasts) were analyzed, with 1724 breasts receiving AlloDerm and 1433 breasts receiving DermACELL. The analysis revealed that AlloDerm was associated with a slightly higher incidence of seroma formation (risk ratio = 1.49, 95% CI, 1.14-1.95, *P* = .003) compared with DermACELL, which may be because of surgical technique rather than ADM. No significant differences were observed in the rates of all other complications and BREAST-Q outcomes. Further, there was no statistically significant difference in the rate of red breast syndrome after sensitivity analysis.

**Conclusions:**

DermACELL demonstrated a slightly lower incidence of seroma formation compared with AlloDerm. Further randomized trials are needed to confirm these results and explore the long-term outcomes associated with both ADMs.

**Level of Evidence: 3 (Therapeutic):**

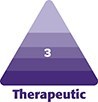

Breast reconstruction is a pivotal component of postmastectomy care, significantly influencing the physical and psychological recovery of patients with breast cancer.^1,2^ The choice of materials and techniques in breast reconstruction is critical to achieving optimal outcomes. Acellular dermal matrices (ADMs) are becoming more commonly used in breast reconstruction because of their potential to provide implant structural support and decrease complication risk, thereby potentially improving aesthetic outcomes.^3,4^ Among the various ADMs available in the United States, AlloDerm (LifeCell, Branchburg, NJ) and DermACELL (LifeNet Health, Virginia Beach, VA) are widely used and have been the focus of numerous clinical studies.^5-16^

AlloDerm and DermACELL originate from the same human cadaver tissue source but are treated with unique, company-specific methods.^15^ For AlloDerm, the process starts by utilizing a buffer to separate the outer skin layer (epidermis) from the underlying tissue. Then, a series of gentle washes removes all cells without altering the tissue's structure.^15^ Patented freeze-drying technology is employed to preserve the tissue without damage, followed by electron beam sterilization to achieve a sterility assurance level (SAL) of 10^−3^.^17^ Unlike its earlier version, which needed soaking before surgery, AlloDerm is now supplied in a ready-to-use (RTU) form, making it more convenient for surgical application.

DermACELL is treated using a combination of various solutions. This mixture includes a non-damaging anionic detergent, 3 distinct antibiotics, and a recombinant endonuclease enzyme, which work together to eliminate up to 97% of nucleic acids from the donor tissue.^15^ After processing, the resulting matrix is stored in glycerol at room temperature, a method that eliminates the need for rehydration before surgical insertion. Sterilization for DermACELL is achieved through low-dose gamma irradiation performed at low temperatures, reaching a SAL of 10⁻^6^.^18^

Essentially, the fundamental difference distinguishing these 2 products is the SAL achieved following their respective radiation sterilization processes; in other aspects like size, shape, and intended performance, they are presented as similar. This report details a systematic review and meta-analysis conducted to comprehensively compare the clinical results observed when using either DermACELL or AlloDerm for breast reconstruction. By synthesizing data from many primary studies, we seek to provide a clearer and more objective understanding of the relative benefits and drawbacks of each ADM, thereby guiding clinicians in making informed decisions about their use in breast reconstruction procedures.

Because of the conflicting evidence in the literature, with studies reporting varying rates of complications between these 2 ADMs, a meta-analysis will help in making more definitive conclusions regarding these outcomes, helping optimize patient care and improving overall success rates of breast reconstruction surgeries.

## METHODS

### Study Design and Protocol

To ensure a rigorous and transparent process, the specific plan (protocol) for this study was formally registered on the international prospective register of systematic reviews (PROSPERO ID: CRD42024542928) database beforehand. Furthermore, the research methodology and reporting followed the established Preferred Reporting Items for Systematic Reviews and Meta-Analyses (PRISMA) regulations (Appendix).^19^

### Search Strategy

A literature search was executed using the PubMed, Scopus, and Web of Science databases. This search was carried out on April 30, 2024, using key terms such as (breast) AND ((DermACELL) OR (AlloDerm)). Another identical search was conducted on April 5, 2025.

### Eligibility Criteria

For inclusion, studies needed to be observational (specifically case–control, cohort, or cross-sectional designs) and written in English. They had to focus on adult patients (18 years or older) undergoing breast reconstruction with either DermACELL or AlloDerm, and crucially, they must have reported comparative data on the incidence of complications of both AlloDerm and DermACELL—meaning that all included studies must be head-to-head comparative studies.

Conversely, studies were excluded if they were commentaries, reviews, other systematic reviews or meta-analyses, case reports, case series, or involved animal research. When duplicate publications of the same study were found, the most recent version involving the largest number of participants was selected.

### Screening and Data Extraction

The study selection procedure involved 2 reviewers (J.T. and S.T.) independently assessing articles against these criteria. If they disagreed on a study's eligibility, a third independent reviewer (Y.T.) was consulted to achieve resolution.

Data extraction followed a similar independent, dual-reviewer process (J.T. and S.T.). Two reviewers independently pulled the necessary information from each included study, and their results were compared to ensure accuracy. Again, a third reviewer (Y.T.) resolved any discrepancies. The extracted details for creating baseline and summary information covered the first author's last name, publication year, study design, number of patients and breasts, patient age and BMI, counts of smokers and patients with diabetes mellitus, and the follow-up duration. Outcome data extracted included BREAST-Q patient satisfaction, red breast syndrome, capsular contracture, implant failure, necrosis, wound dehiscence, delayed healing, explantation, infection, hematoma, seroma, and drain time. Any complication reported by at least 3 studies was analyzed, and a corresponding forest plot was generated. Thus, although skin flap necrosis, delayed healing, wound dehiscence, and hematoma may not be ADM-specific complications, they were included because they were reported by most of the included studies and to provide an objectively comprehensive safety profile.

### Quality Assessment

The methodological quality of the included articles was assessed using the Newcastle–Ottawa Scale (NOS). A score of 7 or more on this scale indicated a high-quality study, whereas a score of 6 or less designated it as low quality.^20^

### Statistical Analysis

The statistical analysis of the collected data was performed using Review Manager (RevMan) software, version 5.4. Continuous data points (like measurements or durations) were expressed as mean differences (MDs), whereas dichotomous data (representing yes/no outcomes) were represented by risk ratios (RRs). Both of these statistical measures were presented along with their corresponding 95% CIs to indicate the range of possible true values.

The method for combining study results depended on the degree of variation, known as heterogeneity, between them. A fixed-effects model was applied when this heterogeneity was considered low or insignificant. Conversely, if significant heterogeneity was detected (indicated by a *P*-value of <.05), a random-effects model was employed, which better accounts for the variability between studies. To investigate the impact of individual studies on significant heterogeneity, a sensitivity test called the “leave-one-out” method was used. For all analyses, findings were considered statistically significant if the calculated *P*-value fell below .05.^21^

## RESULTS

### Summary of Studies

**Figure 1.**
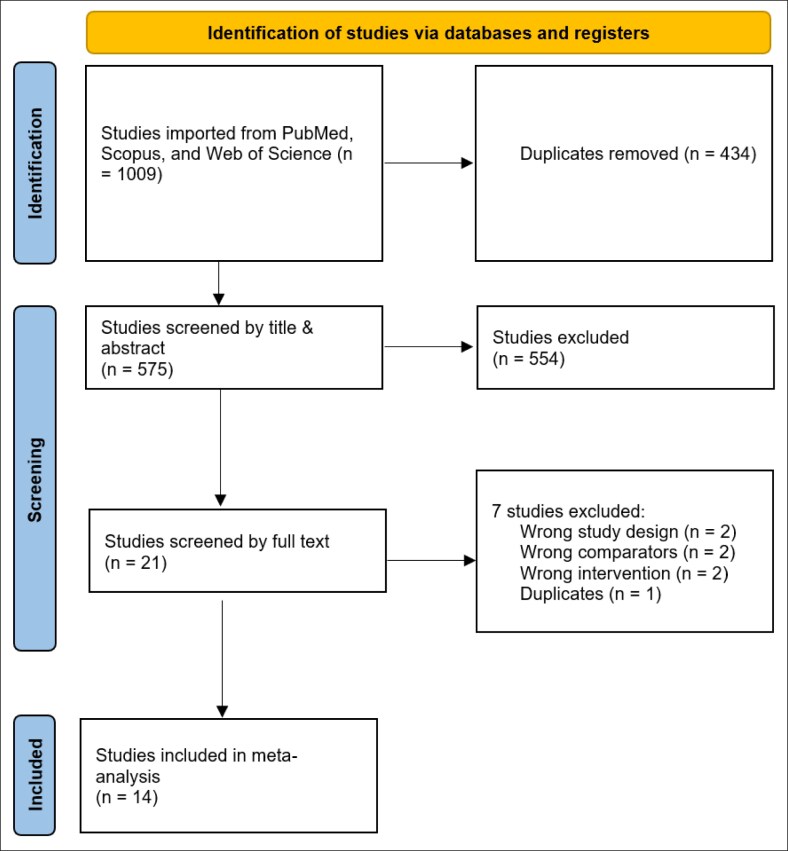
Preferred Reporting Items for Systematic Reviews and Meta-Analyses flow diagram. Flow diagram illustrating the systematic search and study selection process, including the number of records identified, screened, excluded, and included in the meta-analysis.

The literature search process involved 2 phases, conducted first in April 2024 and then updated recently in April 2025. Together, these searches initially identified 1009 potentially relevant studies. After eliminating 434 duplicate records, 575 unique studies proceeded to the title and abstract screening stage. Based on this initial screening, 554 studies were excluded as irrelevant. Consequently, 21 studies were deemed eligible for a detailed full-text review to confirm their suitability. Upon completion of the full-text assessment, 14 studies fully met the inclusion criteria and were incorporated into the meta-analysis.^5-16,22,23^ This step-by-step study selection process is illustrated according to PRISMA in Figure 1.^19^ Notably, 2 of these final included studies were identified during the second search phase carried out on April 5, 2025.^22,23^

Regarding the methodological quality, the assessment revealed that all 14 studies included in this meta-analysis were considered to be of good overall quality according to the NOS.^20^ The detailed evaluation supporting this can be viewed in the Supplemental Table.

The combined patient pool across these studies included a total of 1872 individuals, representing 2940 breasts. Breaking this down by product, the AlloDerm group included 1724 breasts, and the DermACELL group included 1433 breasts.

Baseline characteristics, including age, BMI, smoking status, diabetes, and follow-up duration, were comparable between the AlloDerm and DermACELL groups (Table 1).

**Table 1.** Baseline Characteristics of Patients in Included Studies

Study Author (year)	Study design	No. of patients		No. of breasts		Age (years)		BMI		Smoker		Diabetes mellitus		Follow-up time (months)		Chemotherapy		Radiotherapy	
		AlloDerm	DermACELL	AlloDerm	DermACELL	AlloDerm	DermACELL	AlloDerm	DermACELL	AlloDerm	DermACELL	AlloDerm	DermACELL	AlloDerm	DermACELL	AlloDerm	DermACELL	AlloDerm	DermACELL
Diffley et al (2025)^23^	Retrospective cohort	72	15	122	22	51.4	56.5	28.5	26.8	5	2	12	2	549	471	43 (neo + adj)	4	22 (neo + adj)	0
Berger et al (2024)^22^	Prospective cohort	231	405	351	712	51	49	26.7	23.4	76	105	13	28	25	23.2	125 (neo + adj + both)	186	76	105
Davison et al (2024)^15^	Prospective cohort	55	55	55	55	46.27	46.27	24.87	24.87	NA	NA	NA	NA	NA	NA	29	29	17	22
Asaad et al (2023)^14^	Retrospective cohort	300	29	438	41	50.7	54.2	27	27.2	12	0	15	5	15.3	17.6	78 preop 45 postop	10 preop 5 postop	NA	NA
Johnson et al (2022)^7^	Retrospective cohort	88	62	143	98	48.8	46	25.5	24	5	1	2		NA	NA	29	12	NA	NA
Swisher et al (2022)^12^	Retrospective cohort	61	13	108	25	49.3	48.1	27.8	28	10	2	3	0	5.86	4.6	55	10	20	6
Powers et al (2021)^11^	Retrospective cohort	41	38	NA	NA	49	45	25.4	25.6	NA	NA	2	1	29.4	10.1	10	8	12	12
Stein et al (2020)^9^	Randomized controlled trial	23	27	NA	NA	49	54	25	24	NA	NA	0	1	NA	NA	2	1	2	6
Arnaout et al (2020)^10^	Randomized controlled trial	33	33	41	40	47.8	51.4	24.9	24.9	NA	NA	0	1	NA	NA	4	3	5	8
Greig et al (2019)^5^	Retrospective cohort	28	36	39	56	52.4	53.1	24.3	24.9	0	0	1	0	17.25	18.96	14	17	10	11
Chang and Liu (2017)^13^	Prospective cohort	15	14	22	20	47.5	54.1	25.7	25.7	15	11	NA	NA	15	15	6	8	NA	NA
Zenn and Salzberg (2016)^6^	Retrospective cohort	70	70	130	119	NA	NA	NA	NA	NA	NA	NA	NA	6-24	6-24	NA	NA	NA	NA
Pittman et al (2017)^8^	Retrospective cohort	28	30	50	50	46	47.7	24.1	25.8	0	1	0	0	29.4	10.1	10	8	12	12

The table summarizes the baseline characteristics of patients across studies included in the meta-analysis. Reported variables include mean patient age (years), BMI (kg/m^2^), smoking status (percentage of patients), diabetes status (percentage of patients), and follow-up duration (months). NA, not applicable; preop, preoperative; postop, postoperative.

The included studies reported no statistically significant differences in most of the baseline characteristics, including age or smoking prevalence, between groups. This comparability in patient characteristics supports the validity of outcome comparisons between the 2 ADMs. Additional baseline characteristics for the study populations are detailed in Table 1. It is important to note that the study by Danino et al is not represented in this baseline data table because insufficient information on baseline characteristics was available from that publication.^16^

### Outcomes

#### Red Breast Syndrome

**Figure 2.**
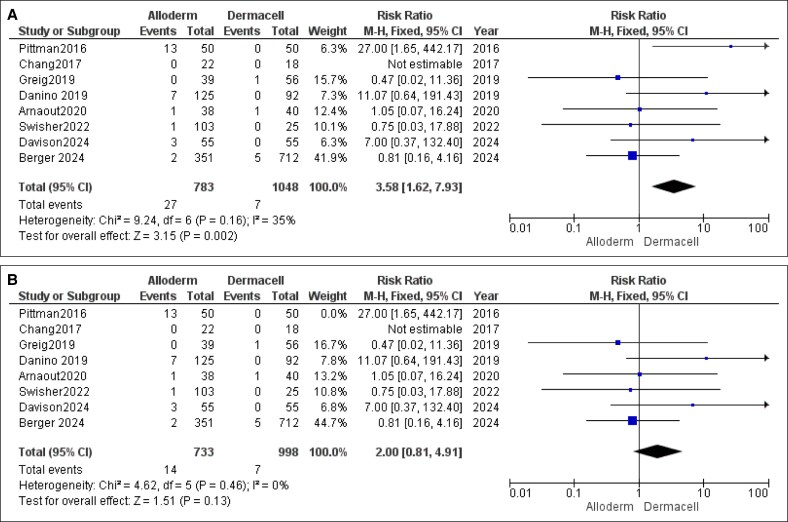
(A) Red breast syndrome forest plot. Forest plot presenting pooled risk ratio (RR) and 95% CIs for red breast syndrome comparing AlloDerm and DermACELL across included studies. Calculated using the Mantel–Haenszel (MH) method under a random-effects model. (B) Red breast syndrome forest plot after sensitivity analysis (excluding Pittman et al). Forest plot showing recalculated pooled risk ratio (RR) and 95% CIs for red breast syndrome after excluding the Pittman et al’s study.

When analyzing the combined data for red breast syndrome, a statistically significant link was found. The results showed that using AlloDerm was associated with a higher rate of this complication compared with using DermACELL (RR = 3.58, 95% CI, 1.62-7.93, *P* = .002). The variation between studies (heterogeneity) for this outcome was low and not significant (*P* = .16, *I*^2^² = 35%), as detailed in Figure 2A. Nonetheless, a post hoc sensitivity analysis excluding Pittman et al revealed no statistically significant association between AlloDerm and a higher rate of red breast syndrome (RR = 2.00, 95% CI, 0.81-4.91, *P* = .13).^8^ Further, the heterogeneity decreased to *I*^2^ = 0% (*P* = .46) indicating that this study may be an outlier, and no true association exists between AlloDerm and red breast syndrome, as shown in Figure 2B.

#### Capsular Contracture

**Figure 3.**
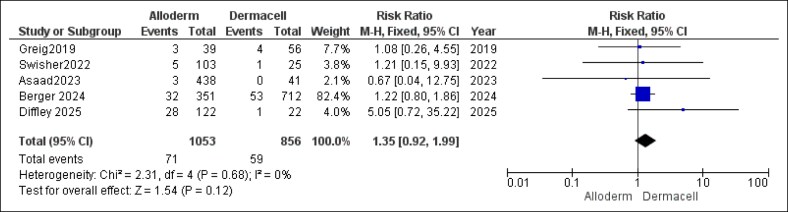
Capsular contracture forest plot. Forest plot displaying pooled risk ratio (RR) and 95% CIs for capsular contracture outcomes comparing AlloDerm and DermACELL.

For capsular contracture, the pooled analysis did not find a statistically significant difference between the AlloDerm and DermACELL groups (RR = 1.35, 95% CI, 0.92-1.99, *P* = .12). No heterogeneity was observed among the studies for this outcome (*P* = .68, *I*^2^ = 0%), as illustrated in Figure 3.

#### Implant Failure

**Figure 4.**
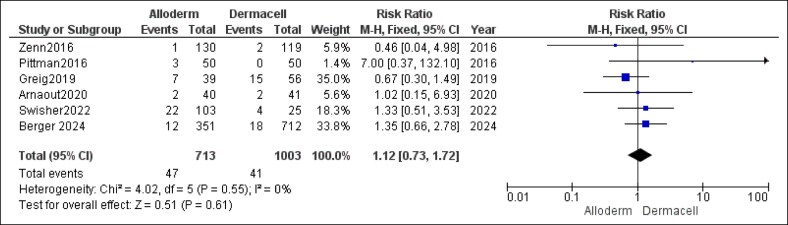
Implant failure forest plot. Forest plot presenting pooled risk ratio (RR) and 95% CIs for implant failure rates.

Similarly, the combined analysis indicated no statistically significant difference in the rates of implant failure when comparing AlloDerm and DermACELL (RR = 1.12, 95% CI, 0.73-1.72, *P* = .61). No heterogeneity was detected (*P* = .55, *I*^2^ = 0%), with details shown in Figure 4.

#### Skin Flap Necrosis

**Figure 5.**
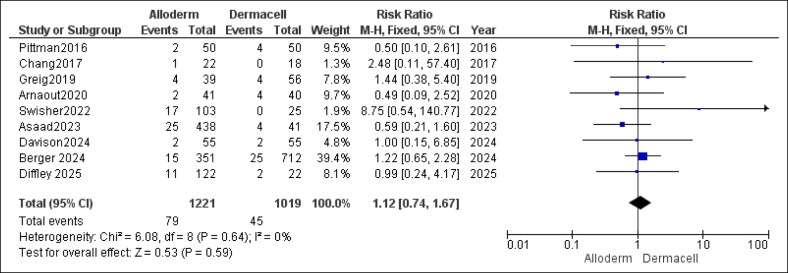
Skin flap necrosis forest plot. Forest plot showing pooled risk ratio (RR) and 95% CIs for skin flap necrosis outcomes.

Regarding the incidence of skin flap necrosis, the pooled results showed no statistically significant difference between AlloDerm and DermACELL (RR = 1.12, 95% CI, 0.74-1.67, *P* = .59). There was no heterogeneity detected (*P* = .64, *I*^2^ = 0%), as depicted in Figure 5.

#### Wound Dehiscence

**Figure 6.**
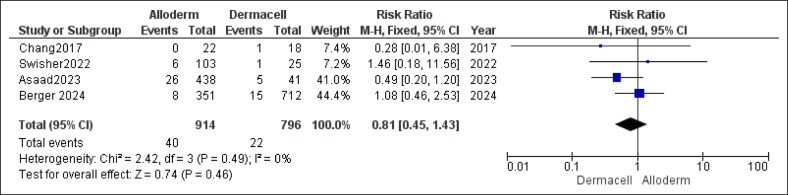
Wound dehiscence forest plot. Forest plot displaying pooled risk ratio (RR) and 95% CIs for wound dehiscence outcomes.

The analysis of wound dehiscence rates did not reveal a statistically significant difference between the 2 groups (RR = 0.81, 95% CI, 0.45-1.43, *P* = .46). Heterogeneity was absent (*P* = .49, *I*^2^ = 0%), as shown in Figure 6.

#### Delayed Healing

**Figure 7.**
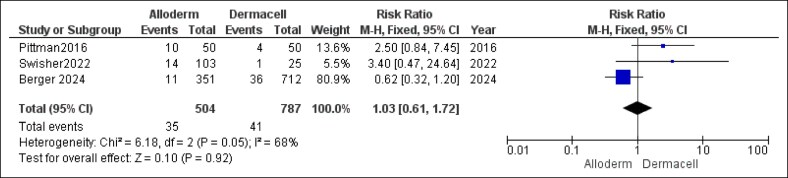
Delayed healing forest plot. Forest plot presenting pooled risk ratio (RR) and 95% CIs for delayed healing outcomes.

For delayed healing, the combined analysis found no statistically significant association between the choice of ADM and the risk (RR = 1.03, 95% CI, 0.61-1.72, *P* = .92). However, statistically significant and substantial heterogeneity was observed among the studies for this outcome (*P* = .05, *I*^2^ = 68%), as illustrated in Figure 7.

#### Explantation

**Figure 8.**
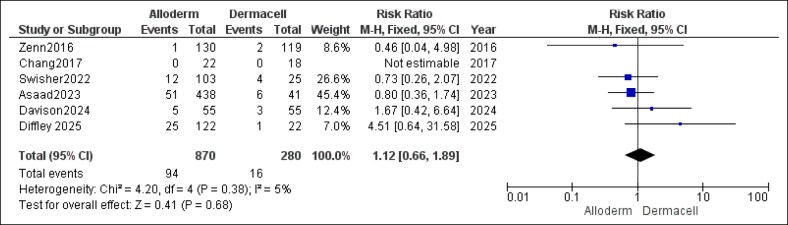
Explantation forest plot. Forest plot showing pooled risk ratio (RR) and 95% CIs for explantation rates (implant removal).

Analysis of explantation rates (implant removal) showed no statistically significant difference between AlloDerm and DermACELL (RR = 1.12, 95% CI, 0.66-1.89, *P* = .68). Heterogeneity was low and not significant (*P* = .38, *I*^2^ = 5%), detailed in Figure 8.

#### Infection Rates

**Figure 9.**
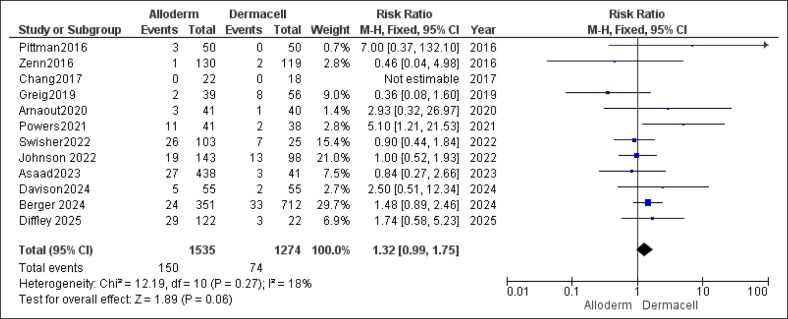
Infection rates forest plot. Forest plot displaying pooled risk ratio (RR) and 95% CIs for infection outcomes.

Regarding infection rates, the pooled analysis indicated no statistically significant difference between the AlloDerm and DermACELL groups, although the trend approached significance (RR = 1.32, 95% CI, 0.99-1.75, *P* = .06) for slightly higher rates with AlloDerm. The heterogeneity was low and not significant (*P* = .27, *I*^2^ = 18%), as shown in Figure 9.

#### Hematoma

**Figure 10.**
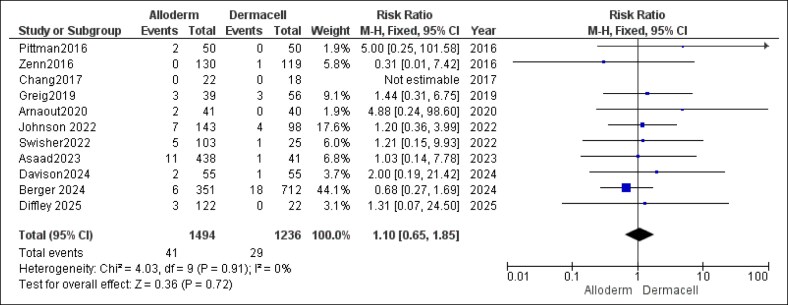
Hematoma forest plot. Forest plot presenting pooled risk ratio (RR) and 95% CIs for hematoma outcomes.

The analysis found no statistically significant difference in hematoma rates between AlloDerm and DermACELL (RR = 1.10, 95% CI, 0.65-1.85, *P* = .72). No heterogeneity was observed (*P* = .91, *I*^2^ = 0%), as illustrated in Figure 10.

#### Seroma

**Figure 11.**
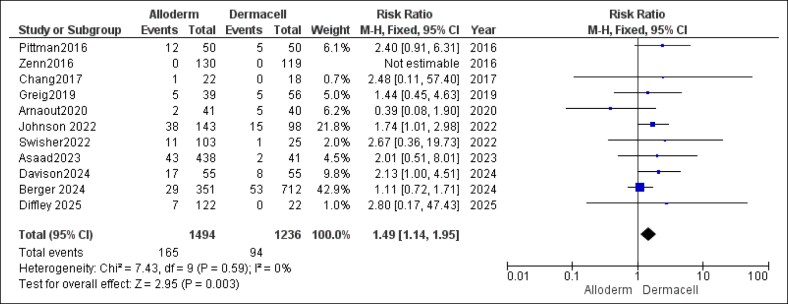
Seroma forest plot. Forest plot showing pooled risk ratio (RR) and 95% CIs for seroma formation.

For seroma formation, the pooled analysis revealed a statistically significant association. AlloDerm was linked to higher rates of seroma compared with DermACELL (RR = 1.49, 95% CI, 1.14-1.95, *P* = .003). Heterogeneity was not observed for this outcome (*P* = .59, *I*^2^ = 0%), as shown in Figure 11.

#### Drain Time

**Figure 12.**
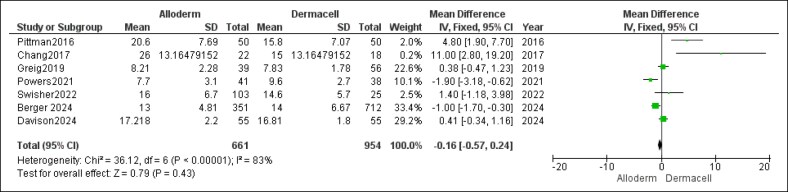
Drain time (days) forest plot. Forest plot displaying mean difference (MD) and 95% CIs for drain duration in days, comparing AlloDerm and DermACELL. Calculated using the inverse variance (IV) method under a random-effects model.

When analyzing the duration surgical drains were left in place, the pooled analysis showed no statistically significant difference between the AlloDerm and DermACELL groups (MD = −0.16 days, 95% CI, −0.57 to 0.24, *P* = .43). However, considerable and statistically significant heterogeneity was observed among the studies for this outcome (*P* < .0001, *I*^2^ = 83%). This high level of variation could not be resolved through sensitivity analysis, and the results are depicted in Figure 12.

#### BREAST-Q Breast Satisfaction

**Figure 13.**
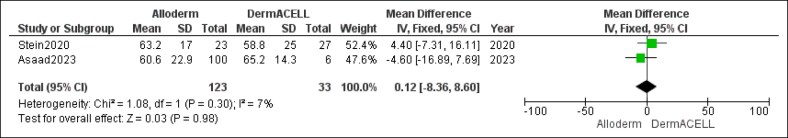
BREAST-Q breast satisfaction. Forest plot presenting mean difference (MD) and 95% CIs for BREAST-Q breast satisfaction scores.

Turning to patient-reported outcomes measured by the BREAST-Q survey, the analysis first examined scores related to breast satisfaction. The results indicated no statistically significant difference in satisfaction levels between patients who received AlloDerm and those who received DermACELL (MD = 0.12, 95% CI, −8.36 to 8.60, *P* = .98). Heterogeneity among the studies for this outcome was low and not significant (*P* = .30, *I*^2^ = 7%), as shown in Figure 13.

#### BREAST-Q Psychosocial Well-Being

**Figure 14.**
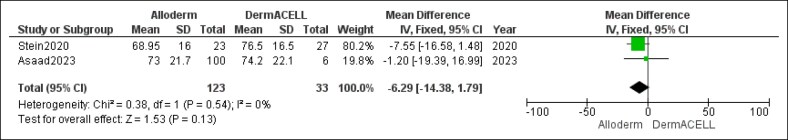
BREAST-Q psychosocial well-being. Forest plot showing mean difference (MD) and 95% CIs for BREAST-Q psychosocial well-being scores.

Next, the analysis looked at BREAST-Q scores concerning psychosocial well-being. Similar to breast satisfaction, no statistically significant difference was found between the AlloDerm and DermACELL groups in this domain (MD = −6.29, 95% CI, −14.38 to 1.79, *P* = .13). No heterogeneity was observed for this outcome (*P* = .54, *I*^2^ = 0%), with details presented in Figure 14.

#### BREAST-Q Sexual Well-Being

**Figure 15.**
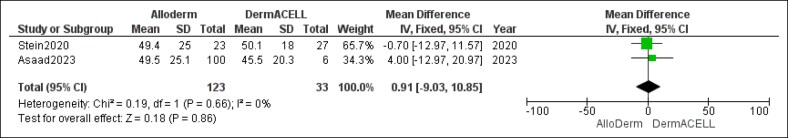
BREAST-Q sexual well-being. Forest plot displaying mean difference (MD) and 95% CIs for BREAST-Q sexual well-being scores.

Finally, the analysis assessed BREAST-Q scores for sexual well-being. Consistent with the other patient-reported outcomes, no statistically significant difference emerged between the AlloDerm and DermACELL groups (MD = 0.91, 95% CI, −9.03 to 10.85, *P* = .98). Heterogeneity was absent (*P* = .66, *I*^2^ = 0%), as illustrated in Figure 15.

## DISCUSSION

Utilizing ADMs has become increasingly common in the field of breast reconstruction surgery. These materials are employed primarily to offer support during the placement of tissue expanders or implants, with the goal of helping to reduce the likelihood of certain complications. Because of their role in the procedure, it is essential for surgeons to possess a clear understanding of the specific risks and potential benefits tied to the particular ADM product they choose to use for their patients.

The variation in manufacturing methods for different dermal matrices is thought to influence tissue integration. It is crucial to identify clinical outcomes, because certain biomechanical and biochemical properties of different structures do not always result in better clinical outcomes in human scenarios. Our results align with the expectations set by other authors in preclinical assays, where outcomes with DermACELL were similar to those of AlloDerm with some weak data showing a potentially better outcome with DermACELL. For instance, Capito et al demonstrated in a rat model that DermACELL had higher cellular migration, infiltration, and density compared with AlloDerm, and also showed higher vascular formation as early as 7 days.^24^ Whether these differences are advantageous or clinically significant remains undetermined. However, our results suggest that the differences observed in the lab may translate into improved clinical outcomes.

One particularly less understood complication associated with ADMs in implant-based breast reconstruction is red breast syndrome. This inflammatory condition occasionally occurs after ADM use, presenting as localized erythema that resembles cellulitis over the ADM. It is thought to stem from lymphatic disruption and resolves through angiolymphatic regeneration.^25^ Our results initially suggested that DermACELL carried less risk of red breast syndrome when compared with AlloDerm. DermACELL and AlloDerm undergo slightly different sterilization processes achieving different Sterility Assurance LevelsAlloDerm (SAL 10⁻^6^ and SAL 10⁻^3^, respectively). This difference, as highlighted by Pittman et al, suggests a potential mechanism for reducing immunological reactions and lowering complication rates associated with ADMs in breast reconstruction.^8^ Nonetheless, after performing a post hoc sensitivity analysis and excluding Pittman et al, we found no statistically significant difference between the 2 ADMs.^8^ Furthermore, the heterogeneity dropped to *I*^2^ = 0% indicating that this study was an outlier. Thus, the exact impact of these sterilization differences on clinical outcomes and pathophysiology remains unclear and require further research.

Despite the ADMs, they have been found to increase the risk of certain complications in breast reconstruction, such as seromas, which can potentially lead to infections and the loss of the prosthetic.^26,27^ This complication often stems from several factors: the diminished blood supply and inflammatory conditions of the skin flap post mastectomy, the intricate spaces left after surgery, and the introduction of a foreign material, which could harbor contamination and develop a biofilm. Although our study found a higher risk for seromas with AlloDerm compared with DermACELL, this association did not extend to an increased risk of infection between the 2 products. Notwithstanding, the seroma outcome should be cautiously interpreted because seromas are associated with patient factors and surgical technique. Because DermACELL is a newer ADM and surgical techniques have improved with time, this may be a confounding variable contributing to the lower incidence of seroma with DermACELL. An example of variation in surgical technique is the Powers et al’s study which reported a statistically significant difference in the proportion of DermACELL patients undergoing direct-to-implant breast reconstruction compared with the AlloDerm arm—although it is worth noting that all the surgeries were performed by the same surgeon, minimizing potential variation in skill or experience between surgeons.^11^

Although our meta-analysis initially found that DermACELL was associated with lower incidences of red breast syndrome and seroma formation compared with AlloDerm, the Stein et al’s study observed initially lower satisfaction with DermACELL at the 3-month follow-up. This apparent contradiction suggests that factors other than these early complications might influence patient-reported satisfaction in the short term. It is possible that expectations, the nature of patient education, inherent product characteristics not directly related to complications (such as feel or consistency), or other unmeasured clinical practices could play a role. These factors might impact patient perceptions independently of the clinical complication rates.^9^ Furthermore, the resolution of early complications without lasting effects could explain why the differences in patient satisfaction were not observed at the 12-month follow-up in the Stein et al’s study. Patients' initial perceptions could be heavily influenced by their expectations and the immediate postoperative experience, which might be shaped by factors beyond just the complication rates.

Despite the small differences in complication rates observed between DermACELL and AlloDerm, patient-reported outcomes remained similar for both ADMs. This indicates that higher risks of specific complications with 1 ADM do not necessarily translate to lower patient satisfaction. This can be attributed to the effective management and transient nature of these complications, which do not have a long-term impact on patient satisfaction and quality of life. Consequently, even if an ADM has a higher incidence of complications like red breast syndrome or seroma formation, these factors might not significantly detract from the overall positive outcomes as perceived by patients, especially if not further complicated with other entities such as infections.

In the clinical trial conducted by Arnaout et al, they evaluated the effectiveness of DermACELL compared with AlloDerm-RTU in terms of drain duration and other major and minor complications post mastectomy with implant-based breast reconstruction.^10^ Their study did not find statistically significant differences in drain duration or most postoperative complications, including seroma formation, red breast syndrome, infection, capsular contracture, and implant failure. In contrast, our meta-analysis, which aggregated data from 14 studies involving 1872 patients (2940 breasts), provides a broader perspective on the performance of these ADMs in clinical settings. We found that DermACELL was associated with small yet statistically significant lower rates of seroma formation compared with AlloDerm. Interestingly, although not statistically significant, Arnaout's trial reported higher infection rates and more frequent surgical revisions in the AlloDerm-RTU group, suggesting a trend that supports our findings of lower complication rates with DermACELL.^10^

In terms of PROMs and in contrast with our study, where we found no differences in BREAST-Q outcomes, a clinical trial by Stein et al found that AlloDerm showed short-term improvements in patient satisfaction at 3 months postoperatively. However, these differences were not sustained at 12 months, aligning with our results of consistent patient-reported satisfaction for both ADMs over the long term.^9^

Swisher et al investigated outcomes between DermACELL and AlloDerm in breast reconstruction and found no significant differences in complication rates, including implant failure, skin necrosis, hematoma, and red breast syndrome, with the latter showing only a trend toward significance.^12^ Their study included fewer studies, which may influence the breadth of data analysis. In comparison, our analysis, which included 14 studies and a larger cohort of 1872 patients (2940 breasts), initially identified a statistically significant higher incidence of red breast syndrome with AlloDerm compared with DermACELL AlloDerm. This outcome highlights a potential difference in the occurrence of red breast syndrome between the 2 ADMs, contrary to the nonsignificant trend reported by Swisher et al.^12^ It is important to note that although this difference was rendered statistically insignificant after sensitivity analysis with exclusion of the Pittman et al’s study, this study was also included in Swisher’s analysis, and yet they did not find a statistically significant difference, possibly because of their small sample of 3 studies for the red breast syndrome outcome (vs 8 studies in our analysis of this outcome).^8^

Although our study provides valuable insights, it has limitations inherent to meta-analyses, such as potential publication bias and the heterogeneity of included studies in terms of patient populations and surgical techniques, with some studies placing the implant and ADM with a flap, which could potentially result in confounding, especially given that the majority of included studies are observational.^13,28^ Other potential confounders include dates of surgeries (with DermACELL potentially being more commonly used after improvements in surgical technique because of its later release in 2011 compared with AlloDerm which was first used in 1992), breast cup size, specific details about implants including volume, whether implants were part of a direct-to-implant procedure or used with tissue expanders, or postoperative treatments like radiation, which is known to increase complications in implant-based breast reconstruction by 2.6 times.^29-31^ These factors can significantly affect outcomes and should be considered in interpreting our findings. Moreover, although the impact of chemotherapy has not been shown to increase complications, its role in breast reconstruction using ADMs remains unclear.^32^

Future research should focus on conducting randomized controlled trials (RCTs) comparing these 2 ADMs, because this would provide higher quality evidence of their relative efficacies. Such studies should aim to control for or explicitly measure the impact of the aforementioned variables to better understand their effects on the efficacy and safety of ADMs in breast reconstruction.

## CONCLUSIONS

This analysis suggests that DermACELL may be associated with potentially more favorable outcomes regarding the rate of seroma formation compared with AlloDerm. Nonetheless, these findings should be cautiously interpreted because of the inclusion of mostly observational studies and several confounding factors that may not have been controlled for in these included studies. For all other outcomes, no statistically significant differences were found between the 2 ADMs. Therefore, although these findings provide more objective insights than the individual primary studies included in this analysis, further investigation, especially through larger RCTs, is recommended.

## Supplementary Material

ojaf084_Supplementary_Data
